# Supplementary material for: Vitamin D status in breast cancer cases following chemotherapy: A pre and post observational study in a tertiary hospital in Yogyakarta, Indonesia
Source: PLoS One. 2022 Jun 24;17(6):e0270507. doi: 10.1371/journal.pone.0270507 (PMC9231732; doi:10.1371/journal.pone.0270507)
Supplement: S1 Table — Abbreviation: IR: interquartile range; BMI: body mass index; ER: estrogen receptor; PR: progesterone receptor; HER2: human epidermal growth factor receptor 2. (PDF) [file pone.0270507.s001.pdf]

S1 Table. Comparison of baseline vitamin D level among different socio-demographic factors and clinicopathology characteristics (n =136)

Predictors	Baseline vitamin D (ng/ml; median±IR)	p-value
Menopause		0.344
Menopause	8.62±5.33	
Pre-menopause	8.18±4.46	
Parity		0.068
Nullipara	9.78±6.02	
Primipara	5.69±6.11	
Multipara	8.43±3.85	
Education		0.507
Undereducated	8.21±3.50	
Well educated	8.67±5.45	
Occupation		0.837
Housewives	8.45±4.23	
Workers	8.49±4.89	
Insurance		0.994
Underprivileged insurance	8.06±4.73	
Private insurance	8.50±4.51	
BMI (kg/m^2; median±IR)		0.698
Underweight-normal (<25.00 kg/m2)	8.45±5.31	
Overweight-obese (≥25.00 kg/m2)	8.43±2.84	
Histological type		0.525
Ductal infiltrative	8.45±4.91	
Lobular infiltrative	3.74±5.73	
Others	7.87±5.35	
Grade		0.689
Well-moderately differentiated	8.79±5.56	
Poorly differentiated	8.26±4.72	
Tumor size		0.603
≤T1	8.04±5.68	
T2	7.94±4.53	
T3	8.12±4.82	
T4	9.21±4.31	
Stage		0.911
Stage 1-2	8.43±3.43	
Stage 3	8.31±4.93	
Stage 4	9.68±6.41	
ER		0.081
Positive	8.42±4.77	
Negative	8.51±5.16	
PR		0.289
Positive	8.43±4.64	
Negative	8.51±4.88	
HER2		0.323
Positive	8.59±4.11	
Negative	8.43±4.88	

Abbreviation: IR: interquartile range; BMI: body mass index; ER: estrogen receptor; PR: progesterone receptor; HER2: human epidermal growth factor receptor 2.

Due to missing values, the counts of some variables did not add up to the total.
