# Supplementary material for: Vitamin D status in breast cancer cases following chemotherapy: A pre and post observational study in a tertiary hospital in Yogyakarta, Indonesia
Source: PLoS One. 2022 Jun 24;17(6):e0270507. doi: 10.1371/journal.pone.0270507 (PMC9231732; doi:10.1371/journal.pone.0270507)
Supplement: S2 Table — Abbreviation: IR: interquartile range. (PDF) [file pone.0270507.s002.pdf]

S2 Table. Duration between sample collection and nearest chemotherapy administration (n =136)

Observation points	Time elapsed (days; median±IR)
Baseline	8.00±7.00
Post-treatment	13.00±22.00

Abbreviation: IR: interquartile range.
