# Supplementary material for: Vitamin D status in breast cancer cases following chemotherapy: A pre and post observational study in a tertiary hospital in Yogyakarta, Indonesia
Source: PLoS One. 2022 Jun 24;17(6):e0270507. doi: 10.1371/journal.pone.0270507 (PMC9231732; doi:10.1371/journal.pone.0270507)
Supplement: S3 Table — Abbreviation: IR: interquartile range. (PDF) [file pone.0270507.s003.pdf]

S3 Table. Comparison of vitamin D level among different distances of sample collection from nearest chemotherapy administration (n =136)

Observation points	N	Vitamin D concentration (ng/ml; median±IR)	p-value
Baseline			0.137
≤14 days	110	8.79±4.06	
>14 days	26	7.34±4.10	
Post-treatment			0.158
≤14 days	74	6.12±4.57	
>14 days	62	7.50±4.30	

Abbreviation: IR: interquartile range.
