# Supplementary material for: Vitamin D status in breast cancer cases following chemotherapy: A pre and post observational study in a tertiary hospital in Yogyakarta, Indonesia
Source: PLoS One. 2022 Jun 24;17(6):e0270507. doi: 10.1371/journal.pone.0270507 (PMC9231732; doi:10.1371/journal.pone.0270507)
Supplement: S4 Table — Abbreviation: IR: interquartile range. (PDF) [file pone.0270507.s004.pdf]

S4 Table. Comparison of vitamin D level among different seasons at point of sample collection (n =136)

Observation points	N	Vitamin D concentration (ng/ml; median±IR)	p-value
Baseline			0.078
Dry season	65	8.02±5.36	
Rainy season	71	9.09±3.50	
Post-treatment			0.344
Dry season	58	7.46±5.01	
Rainy season	78	6.45±4.54	

Abbreviation: IR: interquartile range.
