# Supplementary material for: Vitamin D status in breast cancer cases following chemotherapy: A pre and post observational study in a tertiary hospital in Yogyakarta, Indonesia
Source: PLoS One. 2022 Jun 24;17(6):e0270507. doi: 10.1371/journal.pone.0270507 (PMC9231732; doi:10.1371/journal.pone.0270507)
Supplement: S5 Table — Abbreviation: IR: interquartile range; CINV: chemotherapy-induced nausea vomiting. (PDF) [file pone.0270507.s005.pdf]

S5 Table. Comparison of post-treatment vitamin D level among different chemotherapy factors (n =136)

Predictors	N	Post-treatment vitamin D (ng/ml; median±IR)	p-value
Chemotherapy cycles			0.381
≤6 cycle	43	6.47±4.85	
>6 cycle	93	7.32±3.41	
CINV			0.338
None-mild	76	7.22±4.79	
Severe	60	6.38±4.52	

Abbreviation: IR: interquartile range; CINV: chemotherapy-induced nausea vomiting.
