# Supplementary material for: Vitamin D status in breast cancer cases following chemotherapy: A pre and post observational study in a tertiary hospital in Yogyakarta, Indonesia
Source: PLoS One. 2022 Jun 24;17(6):e0270507. doi: 10.1371/journal.pone.0270507 (PMC9231732; doi:10.1371/journal.pone.0270507)
Supplement: Attachment — Submitted filename: Response to Reviewers.docx [file pone.0270507.s007.docx]

Dear Editor,

We are thankful for the positive feedback received from the editorial team, and for the opportunity to respond to the constructive points in our submitted manuscript. Please find below our point-by-point response to the feedback outlining, where relevant, the related changes we have made. We have uploaded revised versions of the manuscript as instructed, including both a clean copy and a track changes version, and additional supplementary files made accordingly.

**Comments**

***Journal requirements***

1. Please ensure that your manuscript meets PLOS ONE's style requirements, including those for file naming. The PLOS ONE style templates can be found at https://journals.plos.org/plosone/s/file?id=wjVg/PLOSOne_formatting_sample_main_body.pdf and https://journals.plos.org/plosone/s/file?id=ba62/PLOSOne_formatting_sample_title_authors_affiliations.pdf

**Response**

Thank you for your remark. We have checked these requirements prior to submitting the revised manuscript.

2. Thank you for stating the following financial disclosure: “SHH received funding from Kementrian Riset, Teknologi dan Pendidikan Tinggi Republik Indonesia (ID) (2018) and Universitas Gadjah Mada (2020).” Please state what role the funders took in the study. If the funders had no role, please state: "The funders had no role in study design, data collection and analysis, decision to publish, or preparation of the manuscript." If this statement is not correct you must amend it as needed. Please include this amended Role of Funder statement in your cover letter; we will change the online submission form on your behalf.

**Response:** We confirm that this study only received funding from the stated institutions and the funder indeed had no role in the study design, data collection and analysis, decision to publish, or preparation of the manuscript. We also included the statement in our cover letter.

3. In your Data Availability statement, you have not specified where the minimal data set underlying the results described in your manuscript can be found. PLOS defines a study's minimal data set as the underlying data used to reach the conclusions drawn in the manuscript and any additional data required to replicate the reported study findings in their entirety. All PLOS journals require that the minimal data set be made fully available. For more information about our data policy, please see <http://journals.plos.org/plosone/s/data-availability>. Upon re-submitting your revised manuscript, please upload your study’s minimal underlying data set as either Supporting Information files or to a stable, public repository and include the relevant URLs, DOIs, or accession numbers within your revised cover letter. For a list of acceptable repositories, please see http://journals.plos.org/plosone/s/data-availability#loc-recommended-repositories. Any potentially identifying patient information must be fully anonymized. Important: If there are ethical or legal restrictions to sharing your data publicly, please explain these restrictions in detail. Please see our guidelines for more information on what we consider unacceptable restrictions to publicly sharing data: http://journals.plos.org/plosone/s/data-availability#loc-unacceptable-data-access-restrictions. Note that it is not acceptable for the authors to be the sole named individuals responsible for ensuring data access. We will update your Data Availability statement to reflect the information you provide in your cover letter.

**Response:**

We have provided the minimal data set that were used to generate all tables and supplementary tables demonstrated the findings presented in the paper. The full de-identified dataset would not be able to be shared publicly due to restrictions imposed by the ethics committee as most of these are contain patient data, albeit de-identified, and it may be possible to determine the identify of participants given the extent of sociodemographic and clinical data available for each participant.

Should there be a request for data, this can be sent to the corresponding author (email: susanna.hutajulu@ugm.ac.id). Future researchers can contact the institutional ethics committee (email: mhrec_fmugm@ugm.ac.id) at Universitas Gadjah Mada, Indonesia, with data access queries as well.

In the revised manuscript we have uploaded the minimal data set as Supporting Information file. We also restated our data availability statement in the cover letter.

4. Please ensure that you refer to Figure 1 in your text as, if accepted, production will need this reference to link the reader to the figure.

**Response:** Thank you for your suggestion, the flow of subjects’ enrolment in the study as presented in Figure 1 is now cited in line 196.

***Additional Editor comments***

In order to address the review in the best possible way, the following aspects should be clarified

1. How was the sample size calculated?

**Response:**

Thank you for your question about the sample size calculation used in the study. The sample calculation was performed to assess the main outcome in the study, observing changes in post-treatment vitamin D level. The minimal sample size was calculated to compare two paired means using paired t-test analysis, using standard deviation of differences obtained from previous study by Gabr and Marei (2017). We calculated the minimal sample to present 90% power with 5% significance. Although the minimal sample size required is 119 subjects, all subjects meeting the inclusion criteria from the main study is included to increase the power of data presented, making the final number of samples of the study into 136 subjects. We have now added in lines 164-166 under Methods.

2. Define the "baseline" time for measuring the basal levels of vitamin D.

**Response:**

In this study, baseline blood sampling was taken within the time of subjects’ diagnosis until prior to the first dose of chemotherapy administration. The proposed timeline was within one week. However, during COVID 19 pandemic this timeline was altered. In some cases chemotherapy initiation was postpone while baseline blood sampling has already been performed, resulting in longer time space between sampling and chemotherapy administration. Same problem occured for post chemotherapy blood sampling when patients did not visit hospital in their planned schedule. Sampling timeline ranged 0-147 days before first chemotherapy and 3-125 days after the last chemotherapy. We now have made the definition of the baseline observation in the study clearer. It is now added in lines 144-150 under Methods.

3. Provide the coefficients of variability for the measurement of serum 25-OH-D.

**Response:**

Thank you for your query on the coefficients of variability in the study. The intra- and inter-assay coefficient of variability (%CV) was 4.7% and 10.2%, respectively. This information is now disclosed in lines 155-156 under Methods.

4. Avoid repeating the same results in the text and table.

**Response:**

Thank you for this comment. We have reviewed our manuscript and made necessary adjustments to avoid repetition of the results already presented in all tables and modified Fig 1 legend.

5. Indicate in the tables (example table 2) if statistical tests have been performed between different groups and if the test is significant (eg vitamin D among nullipara, primipara and multipara).

**Response:**

Thank you for your suggestion. In this study, we compared the mean vitamin D between groups using independent t-test and ANOVA test and found no significant difference on all variables observed. We have added this information accordingly under Methods in lines 169-172 and legends of Table 2 and Table 4. We have also added the result of between groups comparison of baseline vitamin D under Results in lines 236-238, citing the corresponding results presented as S1 Table, and comparison of post-chemotherapy vitamin D level in lines 288-290, citing S5 Table.

6. Analyze in more detail the differences between baseline and post-chemotherapy using median value and interquartile range. Also statistically define the difference.

**Response:**

Thank you for your advice to convey the differences of vitamin D level in more details. Under Results in lines 255-258, we explained that the paired t-test analysis found a significant change of the vitamin D concentration, as compared to the level measured at baseline (p <0.001). The median and interquartile value obtained from computing the absolute changes of vitamin D between the two observation points was 3.13±4.03 ng/ml.

7. Consider the time elapsed between baseline and final dosing and assess whether the seasonality of the sampling may have influenced the result.

**Response:**

We have performed additional analysis to evaluate the time elapsed between sample collection and the closest chemotherapy completion, as well as the seasonality of the samples in our study. The time elapsed was calculated as the days between the sample collection and the closest last chemotherapy administration to the subjects. For the seasonality analysis, we matched the date of the sample collection with the season as determined by the national Meteorological, Climatological, and Geophysical Agency for the area of Yogyakarta, where the study took place. The analysis was performed using independent t-test as described under Methods in lines 171-175 and presented in S3 and S4 Tables as cited under Results in lines 265-271.

We hope that, following our revisions, the manuscript is now suitable for publication in the PLOS ONE.

Yours sincerely,

Susanna Hutajulu, MD, PhD
