# Supplementary material for: Vitamin D status in breast cancer cases following chemotherapy: A pre and post observational study in a tertiary hospital in Yogyakarta, Indonesia
Source: PLoS One. 2022 Jun 24;17(6):e0270507. doi: 10.1371/journal.pone.0270507 (PMC9231732; doi:10.1371/journal.pone.0270507)
Supplement: Attachment — Submitted filename: Response to Reviewers.docx [file pone.0270507.s008.docx]

Dear Editor and Reviewer,

We are thankful for the positive feedback received from the reviewer, and for the opportunity to respond to the constructive points in our submitted manuscript. Please find below our point-by-point response to the feedback outlining, where relevant, the related changes we have made. We have uploaded revised versions of the manuscript as instructed, including both a clean copy and a track changes version, and additional supplementary files made accordingly.

***Reviewer #1***

1. The title is OK

**Response**

Thank you for your feedback.

2. In abstract: the objective differs from that of the text.

**Response:**

The objective in the Abstract previously stated as “to observe pre- and post-treatment vitamin D level and factors that influence its changes after chemotherapy in breast cancer patients”, is now changed into “to observe pre -and post-treatment vitamin D level and its association with treatment and concomitant factors in breast cancer patients treated with chemotherapy” (lines 28-30)”

3. In introduction is ok.

**Response:**

Thank you for reviewing this section.

4. In methods: Has the main study been published?

**Response:**

Thank you for your query on the main study. The prospective cohort is still ongoing, thus main data are still under collection. Nested studies exploring diagnostic delay in breast cancer (Hutajulu SH et al., PLoS One 2021) and patients’ experience (Prabandari et al., The Breast 2022) have been published as part of the main study.

5. In methods: Better describe the inclusion and exclusion criteria in the present study.

**Response:**

Thank you for your advice to elucidate the inclusion and exclusion criteria employed in the study. We try to make clear that the subjects included in our study are selected from an ongoing cohort with already selected criteria. Under Methods in lines 98-109, we now explain that “We performed a nested pre-post observational study assessing 25(OH)D changes on primary breast cancer patients registered in a prospective ongoing cohort. The main study analysed the risk of chemotherapy side effects and its effect on survival and quality of life in breast cancer patients. Subjects of the main study were histologically confirmed female breast cancer patients aged ≥18 years who were chemotherapy naïve and receiving their first chemotherapy in the Haematology and Medical Oncology Division, “Tulip”/Integrated Cancer Clinic, Dr. Sardjito General Hospital, Yogyakarta, Indonesia, from 2018-2022. No subjects in the cohort were in a terminal condition or with severe congestive heart failure. Among subjects enrolled in the main cohort, we only included those who have received their last chemotherapy cycle and completed post-treatment follow-up into the present study. Subjects who have incomplete blood sample were excluded.”

6. In methods: Describe the sample size calculation

**Response:**

Thank you for this suggestion. We performed the calculation of sample size to achieve the minimum number of samples for the main outcome. The minimal sample size was calculated for paired mean comparison using paired t-test analysis. The standard deviation of differences was obtained from a previous study by Gabr and Marei (2017), who found the mean difference between pre- and post-treatment vitamin D in Egyptian breast cancer patients to be 5.51 ng/ml (SD=6.67 ng/ml). We calculated the minimal sample to present 90% power with 5% significance. Although the minimal sample size required is 119 subjects, all subjects meeting the inclusion criteria from the main study are included to increase the power of data presented, making the final number of samples of the study 136 subjects. The sample size calculation is briefly explained under Methods in lines 170-172. We have also added the referenced study as a reference [26].

7. In methods: What is the time between the first and second serum vitamin D dosage?

**Response:**

Thank you for your question. We would like to clarify that no vitamin D supplementation was provided to the subjects as part of our study. However, we do perform the vitamin D measurement in two observation points, prior to- and after chemotherapy. The median duration between pre- and post-treatment sample collection in our study was 5.00±1.64 months. Although vitamin D changes between observation points are lower on samples taken more than 5 months apart (median±IR =2.46±3.31 ng/ml) as compared to less than 5 months (median±IR =3.59±4.12 ng/ml), we found that the difference is not statistically significant (p =0.055). While no significant difference in vitamin D concentration was found based on the duration of the delay in collecting blood samples (S3 Table), this delay prolongs the distance between pre- and post-treatment observation and might not represent the actual duration of the chemotherapy received by subjects. Instead, we report our findings by stratifying the vitamin D changes based on the number of chemotherapy cycles administered to better represent the neurotoxicity presumed to cause the decrease of vitamin D concentration in subjects.

8. In results: Age factor is important in terms of serum vitamin D values. What is the age range among the participants? How many women were under 40 years of age?

**Response:**

We agree that age is very important in regards to vitamin D levels. In our study, the median subjects’ age is 51±13 years old. The range of the age is 46, the youngest participant was 32 and the oldest was 78 years old. Based on the Spearman analysis result as presented in Table 2, age did not correlate with the baseline vitamin D level in our study (r =0.096, p =0.266). After stratifying based on the median age, the vitamin D level remains similar among younger and older subjects, both at baseline (p =0.428) and post-treatment (p =0.755). In our study, only 12 (8.82%) subjects were under 40 years old. Vitamin D however remains similar among groups under 40 and older (p =0.753). This result is added under Results in lines 248-249.

9. In results: How long is post-menopause?

**Response:**

Although 72 subjects were already menopause at the beginning of the study, only 67 (93.1%) were able to recall or have any documented examination results of their menopause. Among them, the median age of menopause is 50±6 years old. The median duration of menopause is 7.91±10.5 years when baseline vitamin D was measured. This result is now added under Results in lines 210-213.

10. In results: What is the impact of weight on serum vitamin D values? Because 30% of women were obese.

**Response:**

In our study, the low vitamin D level was observed in all subjects, and no difference was found among BMI categories. The median vitamin D did not differ (p =0.819) among subjects who were underweight (median±IR =8.06±5.44 ng/ml), normal (median±IR =9.10±5.27 ng/ml), overweight (median±IR =9.68±3.16 ng/ml), and obese (median±IR =8.23±2.82 ng/ml). However, we did not observe the body fat distribution of the subjects in our study. The higher proportion of visceral adipose tissue despite lower BMI in the Asian population and its stronger correlation with vitamin D instead of the overall body fat might explain the low vitamin D across different BMI as found in our study. This is now added under Results in lines 250-253 and Discussion in lines 369-372.

11. In results: In table 2, the presentation of the results is not clear.

**Response:**

Thank you for your remarks. In Table 2, we try to present the result of the Spearman correlation analysis result among the socio-demographic and clinicopathological characteristics of the subjects with their baseline vitamin D concentration. The value of baseline vitamin D, as well as the variable age, age menarche, age of first pregnancy, upper arm circumference, Hb, albumin, leucocyte, AST, and ALT, were analysed as continuous data. The variable menopause, parity, education, occupation, insurance, BMI, histological type, grade, tumor size, stage, ER, PR, and HER2 were analysed as categorical data, with the median vitamin D concentration of each category also presented in the table. The result is presented in Spearman rho and p-value. Among the factors observed, only AST shows a significant correlation. In addition to this analysis, we also compare the median vitamin D levels among different categories in each group using an independent t-test and ANOVA test. This result is presented in S1 Tables.

12. In results: In tables 2, 3 and 4, add statistical analysis with p value

**Response:**

Thank you for your advice. We now have added the statistical analysis corresponding to each p-value reported in Tables 2, 3, and 4.

13. In discussion: Could the type of test (ELISA) that was used for serum levels of vitamin D have influenced such low vitamin D results?

**Response:**

Thank you for expressing this concern. We choose the ELISA method to perform vitamin D analysis in our study due to its high availability in our country and to increase its reproducibility and the feasibility to apply the methods directly to clinical service in our setting. The kit we selected has high specificity (74.7% for 25-OH-D2 and 100% for 25-OH-D3) and good agreement with Diasorin LIAISON chemiluminescent immunoassay (CLIA) (R =0.919) and Roche Cobas (ECLIA) (R =0.948) in measuring vitamin D concentration. The respective assays have high correlations with liquid chromatography-tandem mass spectrometry (LC-MS/MS) (R =0.9455 and R =0.9102), which currently has the highest sensitivity and specificity in vitamin D measurement.

In performing the assay, we used low and high control in duplicates on each plate. The range of low and high control provided by the kit was 7.28-15.1 ng/ml and 37.9-78.7 ng/ml, respectively. In every plate, the low (ranged 7.02-11.57 ng/ml) and high (ranged 50.45-55.61 ng/ml) controls measured fell within the QC ranges. In addition, the assay was performed with 0-120 ng/ml standards which were provided in 6 concentrations; 0, 5, 15, 30, 60, and 120 ng/ml. In performing the assay, the lowest sample measured was 0.68 ng/ml and the highest sample measured was 25.16 ng/ml.

All samples were kept in -80 prior to analysis with no history of freeze-thaw. Furthermore, our plasma samples, QC low, QC high, and standards from the kits were treated uniformly and concurrently, minimising any possible treatment biases of the assay. Therefore, the vitamin D concentration of the samples in this study falls within the range of the standard curve and we are confident that the kit could not possibly contribute to the low values of the samples measured.

Considering the similarly low vitamin D concentration observed in Korean breast cancer patients in a study by Kim et al., in 2018 (median =12.94 ng/ml) and the high prevalence of deficiency in various studies in different at-risk Indonesian, the very low vitamin D concentration observed in our study suggest the severity of low vitamin D in Indonesian women. This information about the assay is now added under Methods in lines 170-172 and Discussion in lines 383-393.

**Additional remarks**

In the previous manuscript version, we mislabelled the stratified groups based on subjects’ menopausal status in Table 1. Necessary adjustments have been made in lines 4-5 of Table 1 and under Results in line 207. We confirm that this error does not affect other results presented in the manuscript.

We hope that, following our revisions, the manuscript is now suitable for publication in the PLOS ONE.

Yours sincerely,

Susanna Hutajulu, MD, PhD
